# Expression of Pigment Cell-Specific Genes in the Ontogenesis of the Sea Urchin *Strongylocentrotus intermedius*

**DOI:** 10.1155/2011/730356

**Published:** 2011-07-25

**Authors:** Natalya V. Ageenko, Konstantin V. Kiselev, Nelly A. Odintsova

**Affiliations:** ^1^A.V. Zhirmunsky Institute of Marine Biology, Far Eastern Branch of the Russian Academy of Sciences, Palchevsky Street 17, Vladivostok 690041, Russia; ^2^Institute of Biology and Soil Sciences, FEB RAS, Vladivostok 690022, Russia; ^3^Far Eastern Federal University, Vladivostok 690950, Russia

## Abstract

One of the polyketide compounds, the naphthoquinone pigment echinochrome, is synthesized in sea urchin pigment cells. We analyzed polyketide synthase (*pks*) and sulfotransferase (*sult*) gene expression in embryos and larvae of the sea urchin *Strongylocentrotus intermedius* from various stages of development and in specific tissues of the adults. We observed the highest level of expression of the *pks* and *sult* genes at the gastrula stage. In unfertilized eggs, only trace amounts of the *pks* and *sult* transcripts were detected, whereas no transcripts of these genes were observed in spermatozoids. The addition of shikimic acid, a precursor of naphthoquinone pigments, to zygotes and embryos increased the expression of the *pks* and *sult* genes. Our findings, including the development of specific conditions to promote pigment cell differentiation of embryonic sea urchin cells in culture, represent a definitive study on the molecular signaling pathways that are involved in the biosynthesis of pigments during sea urchin development.

## 1. Introduction

Polyketide compounds are a large group of structurally very diverse multifunctional proteins mainly found in bacteria, fungi, and plants. One of these polyketide compounds, the pigment echinochrome, is synthesized in sea urchin pigment cells in larvae and in adults [1, 2]. These compounds from sea urchins, as well as many marine secondary metabolites, possess highly effective antioxidant, antibacterial, antifungal, antitumor, and psychotropic activities [3–7] and may play a role in immune defense [8].

**Figure 1:**
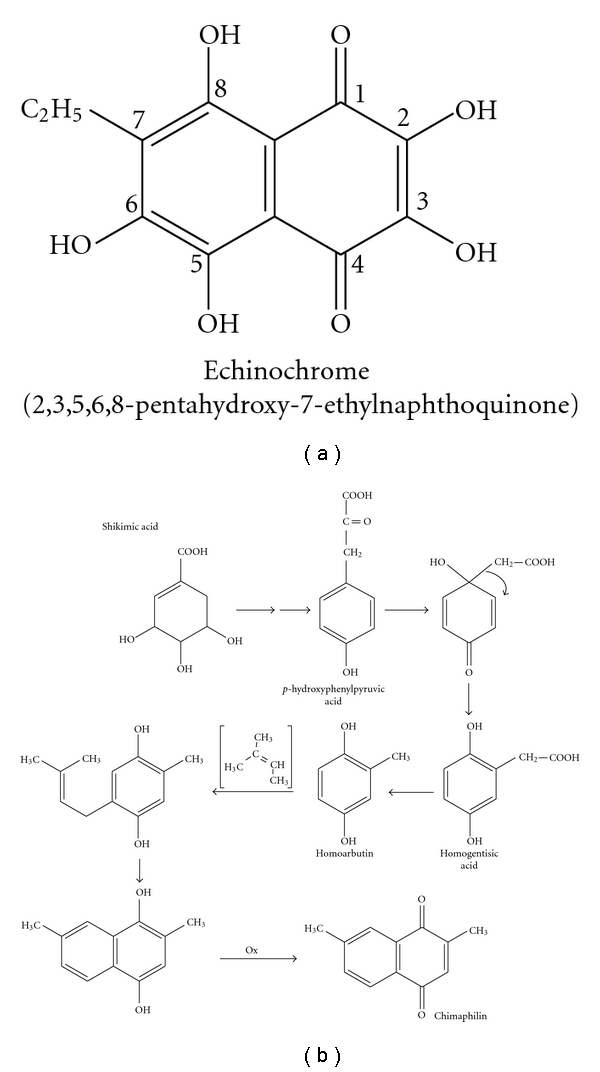
The structure of the naphthoquinone pigment echinochrome (a). One of the quinone biosynthesis pathways (the formation of chimaphilin from a shikimic acid through the mevalonic acid biosynthetic pathway) in accordance with [9] (b).

Although great progress has been made in characterizing sea urchin quinone pigments [1, 2, 9], no definitive information is available on the molecular signaling pathways that are involved in pigment cell specification and the biosynthesis of pigments during sea urchin development. Three basic biosynthetic pathways, the polyketide pathway, the shikimic acid pathway, and the mevalonic acid pathway, are involved in the synthesis of quinones, including benzoquinones, naphthoquinones, anthraquinones, and upper quinones [10]. Different individual compounds are formed by modifications of the basic chemical structure. The bioactive secondary metabolite, echinochrome (2,3,5,6,8-pentahydroxy-7-ethylnaphthoquinone), is in the chemical class of naphthoquinones (Figure 1(a)). It is generated after a series of enzymatic, oxidative, and photochemical reactions from shikimic acid, similar to the formation of chimaphilin through the mevalonic acid biosynthetic pathway, as shown in Figure 1(b).

The drug “histochrome” (registered trademark) was developed from the echinochrome base structure and has unique therapeutic properties [3, 4]. There are three ways to produce echinochrome: aquaculture, chemical synthesis, and the *in vitro* production. The industrial-scale procurement of echinochrome may lead to the extinction of the organisms that produce this substance. Chemically synthesized echinochrome has some toxic effects. Cultured pigment cells of sea urchins could provide a source of pharmacologically important quinone pigments that would help reduce the impact on the adult sea urchin population. The *in vitro* production of biologically active substances is one promising way to solve this problem. 

 Pigment cells are the first type of secondary mesenchymal cells (SMCs) to be specified at the mesenchyme blastula stage in sea urchins [11]. These cells accumulate red-brown pigment granules in their cytoplasm [12] and become easily detectable from the late gastrula stage onwards. The cytoplasmic granules store carotenoids and naphthoquinone compounds [2, 12, 13], which have been suggested to function in body coloring and phototropism which aid in the defense of larval ectoderm [14, 15]. Pigment cell precursors are released from the vegetal plate during the initial phase of gastrulation, and they have the ability to migrate within the ectodermal layer of the larval epithelium [16]. The ability of phagocytosis exhibited by pigment cells suggests their participation in wound healing in larvae [17]. Changes in the normal sequence or rate of sea urchin embryo development affect echinochrome synthesis [18].

Studies have revealed that expression of genes involved in the regulation of embryogenesis and development of sea urchins is mediated by a complex and extended cis-regulatory system [19]. The participation of the sea urchin gene regulatory networks in development has been characterized in detail [20]. The use of the whole mount *in situ* hybridization has revealed that the polyketide synthase (*pks*) gene cluster, three different members of the flavin-containing monooxygenase gene family, and a sulfotransferase gene (*sult*) are specifically expressed in pigment cells, suggesting that they are required for the biosynthesis of the pigment echinochrome [21]. Sea urchin embryos lacking *Sppks* (knock-down) develop pigment cells but appear unpigmented (albino phenotype) [21].

This study is focused on a detailed gene expression profile for two pigment cell-specific genes, *Sipks* and *Sisult*, during sea urchin embryo development and in specific adult tissues. The effect of a precursor of naphthoquinone pigments, shikimic acid, on the expression of pigment cell-specific genes and embryo development was investigated. In addition, specific conditions for promotion of pigment cell differentiation in sea urchin cell culture were developed. The present study is an attempt to increase our understanding of the intracellular mechanisms affecting echinochrome synthesis.

## 2. Materials and Methods

### 2.1. Animals

Adult sea urchins of *Strongylocentrotus intermedius *were collected in the Sea of Japan (Amursky Bay or Vostok Bay) and kept in tubs filled with running, aerated seawater. The animals were rinsed free of any debris with UV-sterilized, filtered seawater and injected with 2-3 mL of 0.5 M KCl to chemically induce spawning. The embryonic material was obtained by artificial fertilization and then placed in tanks with UV-sterilized seawater (17°C) throughout development until the mesenchymal blastula, gastrula, prism, or pluteus stages (14, 24, 34, and 72 hours after fertilization, hpf, resp.). After 48 hpf, the larvae were fed the microalga *Isochrysis galbana *(100 000 cells/mL) daily. The embryos and cell cultures were examined with an inverted microscope Axiovert 200 M (Carl Zeiss, Goettingen, Germany) with 10× and 20× dry objectives.

### 2.2. Real-Time Quantitative Polymerase Chain Reaction (Real-Time PCR)

Quantitative real-time PCR was used to measure the relative amount of *Sppks* and *Spsult *transcripts during the course of development and in specific tissues of the adults. Using BLAST, we showed a high identity (98-99%) of the central part of the *pks* and *sult* genes in the sea urchin *S. intermedius* with that of the *pks *and *sult* genes in the closely related sea urchin *S. purpuratus* (GeneBank accession numbers XM 788471 and DQ176319 for the *pks* and *sult *genes, resp.). Then, we used the obtained nucleotide sequences from cDNA of *S. intermedius* to design the real-time PCR primers and probes. 

Total RNA from spermatozoids, unfertilized eggs, coelomocytes, ambulacra, embryos, and larvae of the sea urchin *S. intermedius* at various stages of development was extracted with Yellow Solve reagent (Clonogen, St. Petersburg, Russia) and treated with DNase. The RNA pellet was washed with 1 mL of 75% ethanol. The sample was then centrifuged at 13,200 g at 4°C for 10 min. Following centrifugation, the supernatant was removed, and the RNA pellet was air-dried and stored at −25°C. For TaqMan real-time RT-PCR, cDNAs were amplified in 20 *μ*L of the reaction mixture containing 1 × TaqMan Buffer B, 2.5 mM MgCl_2_, 250 *μ*M of each deoxynucleotide, 1 U Taq DNA polymerase, 0.5 *μ*L cDNA sample, and 0.25 *μ*M of each primer and probe (Real-Time PCR Kit, Syntol, Russia). Quantitative real-time PCR was performed using the established protocol [22] in the Instrumental Centre of Biotechnology and Gene Engineering of Institute of Biology and Soil Sciences (FEB RAS) using an ABI 310 and 3130 Genetic Analyzers (Applied Biosystems, Foster City, USA). The amplification conditions consisted of one cycle of 2 min at 95°C followed by 50 cycles of 10 s at 95°C and 25 s at 62°C. The TaqMan PCR assays were performed in an iCycler thermocycler supplied with the iQ5 Multicolor Real-Time PCR detection system (Bio-Rad Laboratories, Inc., Hercules, Calif, USA), and data were analyzed with the iQ5 Optical System Software v.2.0 according to the manufacturer's instructions; expression was normalized according to the 2-^ΔΔCT^ method, and the highest scaling option was used (the highest expressing sample was assigned the value 1 in the relative mRNA calculation). The *S. intermedius *actin gene (GenBank accession number DQ229162) was used as an endogenous control to normalize variance in the quality and the amount of cDNA used in each real-time RT-PCR experiment. A nontemplate control for each primer set and a non-RT control (DNase-treated RNA as a template) for each developmental stage were included. No-cycle threshold (Ct) values were consistently obtained after 50 cycles of PCR. The TaqMan probe for the actin gene was labeled with an FAM reporter dye at the 5′-end and an RTQ-1 quencher dye at the 3′-end, and TaqMan probes for the *pks* and *sult *genes were labeled with an ROX reporter dye at the 5′-end and a BHQ-2 quencher dye at the 3′-end (Syntol, Russia). The data were summarized from five independent experiments. The primers 5′GAT CTC CGT CAA CCC ATG AT, 5′CTT GCC CAT GTC ACC ATC,and the probe 5′AAC TAC GGT GTC GAC TCC CTC ATG GC were used for the expression analysis of the *pks* gene. The primers 5′AGA AGC GGC GAA ACA GAA, 5′CCA GAG CCA TTG GTT TTT C, and the probe 5′TGG CGA CTG GAA AAA TCA TTT TAC CGT AGC CCA GA were used for the expression analysis of the *sult* gene. For the actin gene, the primers 5′TGT TGC CCC AGA GGA GCA, 5′ATC TTT TCC CTG TTG GCC TT, and the probe 5′TCC TCC TTA CCG AGG CTC CCC TCA A were used. 

### 2.3. Experiments with Shikimic Acid (ShA)

Sterile solutions of ShA in seawater at the desired concentrations (0.1, 0.5, and 2 mM) were added to sea urchin zygotes (after 20 min pf) and developing embryos at the blastula (14 hpf) and gastrula (24 hpf) stages. Embryos and larvae were cultivated with ShA for 8 days. The development of the culture was monitored to ensure that the embryos were developing normally. After this period, total RNA was isolated from the larvae for the following quantitative real-time PCR. ShA was obtained from Sigma (St. Louis, USA).

### 2.4. Cell Culture

Developing sea urchin embryos were cultivated in 5 L tanks at 17°C and collected on a fine 30 *μ*m nylon mesh at the mesenchymal blastula stage, washed in artificial seawater (Ca^+2^ and Mg^+2^-free salt solution, CMFSS) containing antibiotics (100 IU/mL penicillin and 100 mg/mL streptomycin), and dissociated into single cells with 0.25% collagenase (produced from the hepatopancreas of the red king crab *Paralithodes camtschatica *in the Pacific Institute of Bioorganic Chemistry (PIBOC) of FEB RAS, Vladivostok, Russia) at 17°C (for 20–30 min). The resulting cell suspension containing all cell types was washed several times in seawater with antibiotics, and then sterile seawater supplemented with 2% fetal bovine serum (Sigma) was added. Cell viability was estimated by a trypan blue exclusion test. The cells were seeded at the density of 3 × 10^6^–5 × 10^6^ cells/cm^2^ in plastic Petri dishes (Lux Culture Dishes, ICN Biomedicals), and after two to three days of cultivation, a subset of cells (after several strokes of gentle pipetting) was transferred into new Petri dishes on glass coverslips coated preliminarily with fibronectin (Sigma). The solutions of fibronectin (0.01 mg/mL) were left to settle for 12 h at room temperature (RT). After two washings in sterile seawater, the dishes with the coverslips were stored at RT for 12 to 24 h prior to cell seeding. To cultivate transferred cells, we used two types of the cell culture media: the coelomic fluid preparations of control sea urchins and injured sea urchins. Previously, injured sea urchins were obtained by needle pricks in the area of Aristotle's lantern. Then after a day, the coelomic fluids from 5 control and 5 injured sea urchins were collected by puncture in the area of Aristotle's lantern. After 15–20 min, when the coelomic fluid is taken out of the animal, the coelomocytes aggregated (at 4°C). The coelomic fluid preparations were then centrifuged at 2,300 g (4°C) for 20 min to remove coelomocytes, and the supernatant was sterilized by filtration (0.22 *μ*m, Millipore, USA). The protein content in the supernatants was determined as described previously [23] and averaged 450–475 *μ*g/mL. The cell cultures were maintained by changing the old medium with new medium at 3–5-day intervals for 20 days at 17°C.

### 2.5. Statistical Analysis

Statistical analysis was carried out using the Statistica 8.0 program. The results are represented as the mean ± standard error and were tested by paired Student's *t*-test. *P* < .05 was selected as the point of minimal statistical significance in all analyses.

## 3. Results

### 3.1. Sipks and Sisult Expression Profiles in Sea Urchin Embryo Development and in Specific Adult Tissues

**Figure 2:**
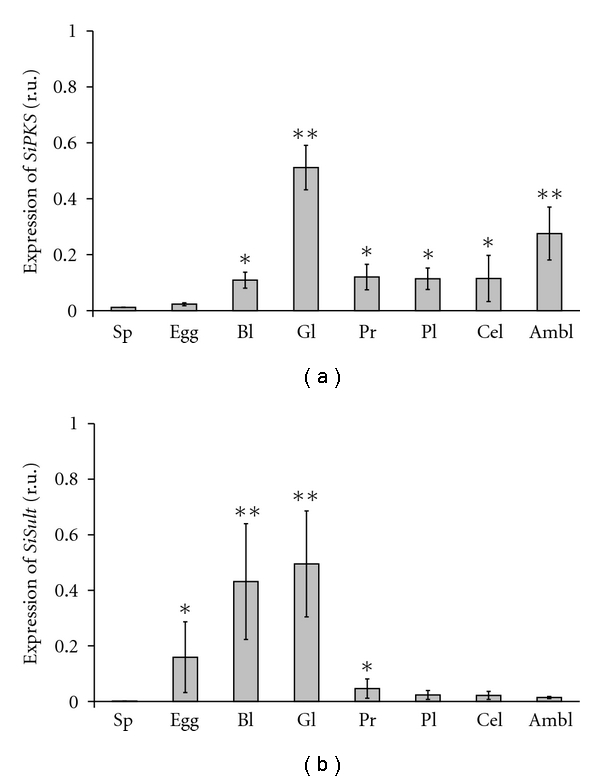
Expression of the *Sipks *(a) and *Sisult* (b) genes in spermatozoids (Sp), unfertilized eggs (Egg), coelomocytes (Cel), ambulacra (Ambl), embryos, and larvae of the sea urchin *S. intermedius* at various stages of development: blastula, 14 hpf (Bl), gastrula, 24 hpf (Gl), prism, 34 hpf (Pr), and pluteus, 72 hpf (Pl). *P < .05; **P < .01.

In unfertilized eggs, only trace amounts of the *pks* and *sult *transcripts were detected, whereas no transcripts of these genes were observed in spermatozoids (Figure 2). We observed the highest level of expression of the *pks* gene at the gastrula stage (Figure 2(a)), which exceeded the expression level of this gene at the blastula, prism, and pluteus stages, and in coelomocytes, and ambulacra by 4.6-, 4.3-, 4.5-, 4.5-, and 1.9-fold, respectively. The gene expression profile for *Sisult *had a similar trend to that of *Sipks. *The onset of transcription for the *sult* gene began at the blastula stage, and then the level of the expression increased drastically through the start of gastrulation (approximately 24 hours) (Figure 2(b)). After that, the level of transcript decreased by more than 10 and 20 times at the prism and pluteus stages, respectively. In addition, *sult *gene expression was detected in coelomocytes and ambulacra, where the level of the *sult* gene expression was lower than that at the gastrula stage by 22.7- and 35.5-fold, respectively.

### 3.2. Experiments with a Precursor of Naphthoquinone Pigments: Shikimic Acid (ShA)

**Figure 3:**
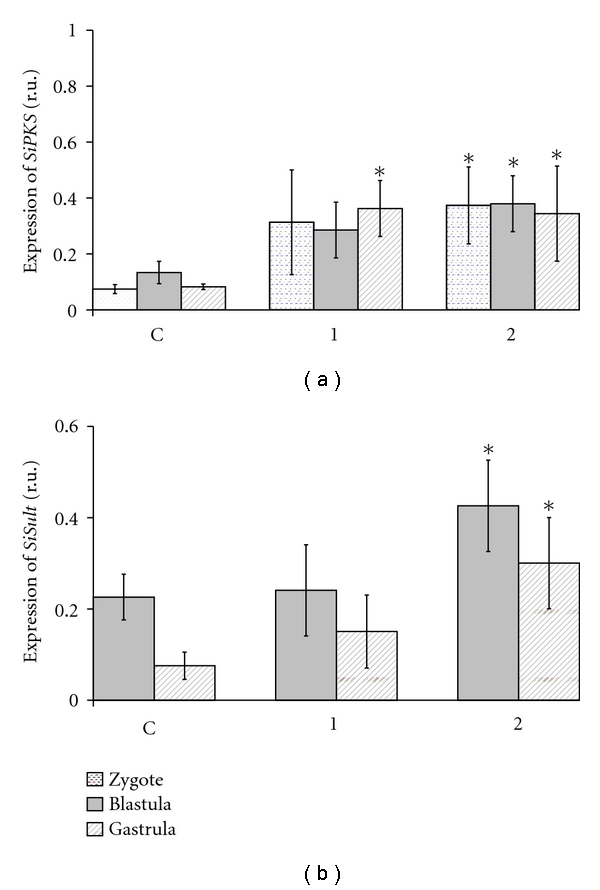
Effect of shikimic acid (ShA) on the expression of the *Sipks *(a) and *Sisult* (b) genes in zygotes, blastula, and gastrula cells of *S. intermedius*. The time of incubation with ShA is 8 days (all embryos of the control group (C) were at the pluteus stage). ShA concentrations tested: 1—0.1 mM, 2— 0.5 mM. *P < .05.

**Figure 4:**
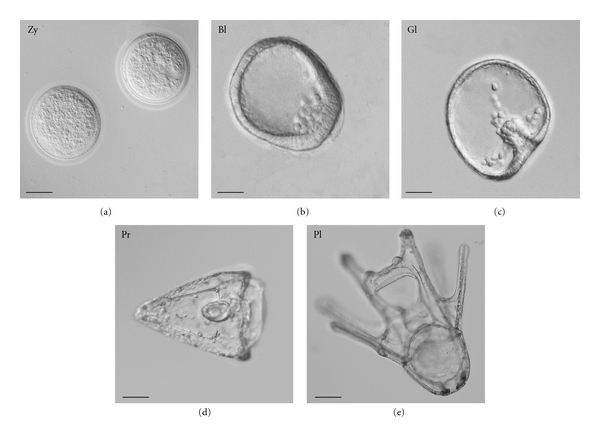
Normal embryo development of the sea urchin *S. intermedius*: zygotes (20 min pf, Zy); blastula stage (14 hpf, Bl); gastrula stage (24 hpf, Gl); (d) prism stage (34 hpf, Pr); (e) pluteus (8 dpf, Pl). Nomarski's optics. *Bar*, 50 *μ*m.

**Figure 5:**
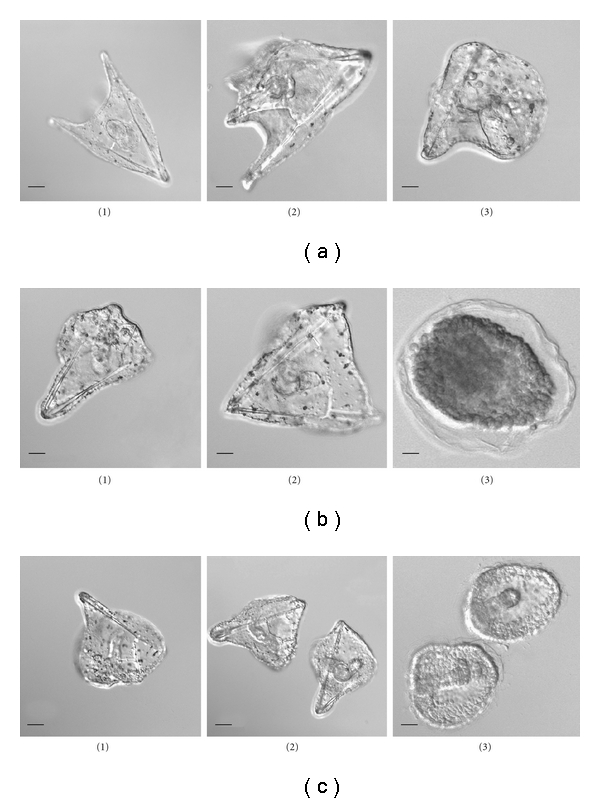
Effect of shikimic acid (ShA) on the larval morphology of the sea urchin *S. intermedius.* Disturbances in embryo development after 8 days of incubation with ShA. ShA was added to (a) zygotes; (b) embryos of the blastula stage; (c) embryos of the gastrula stage. ShA concentrations tested: 1–0.1 mM, 2–0.5 mM, and 3–2.0 mM. Nomarski's optics. *Bar*, 50 *μ*m.

*Sipks* and *Sisult* expression in embryo development was significantly increased after the incubation of sea urchin embryos with 0.1 mM–0.5 mM ShA (Figure 3), but not 2.0 mM ShA, which blocked the expression of the pigment genes (data not shown). No apparent effect on normal development (Figure 4) was detected after the addition of ShA (0.1 mM and 0.5 mM) to sea urchin zygotes, which developed into morphologically almost normal plutei (Figures 5(a)((1), (2))). In contrast, the addition of ShA (0.1 mM and 0.5 mM) to the blastula and gastrula embryos resulted in a marked slowdown of development (Figures 5(b) and 5(c)). In these cases, after 8 days of cultivation with ShA, the development of the sea urchin larvae was retarded in the prism stage, while the control embryos reached the pluteus stage. The addition of 2.0 mM ShA to the zygotes, blastula, and gastrula embryos led to significant disturbances in normal development which was clearly delayed or arrested (Figures 5(a)(3), 5(b)(3), and 5(c)(3)). After the incubation with 2.0 mM ShA, the embryos from the blastula and gastrula stages remained spherical in shape for up to 8 days of development. 

### 3.3. Differentiation of Pigment Cells in Cell Culture

**Figure 6:**
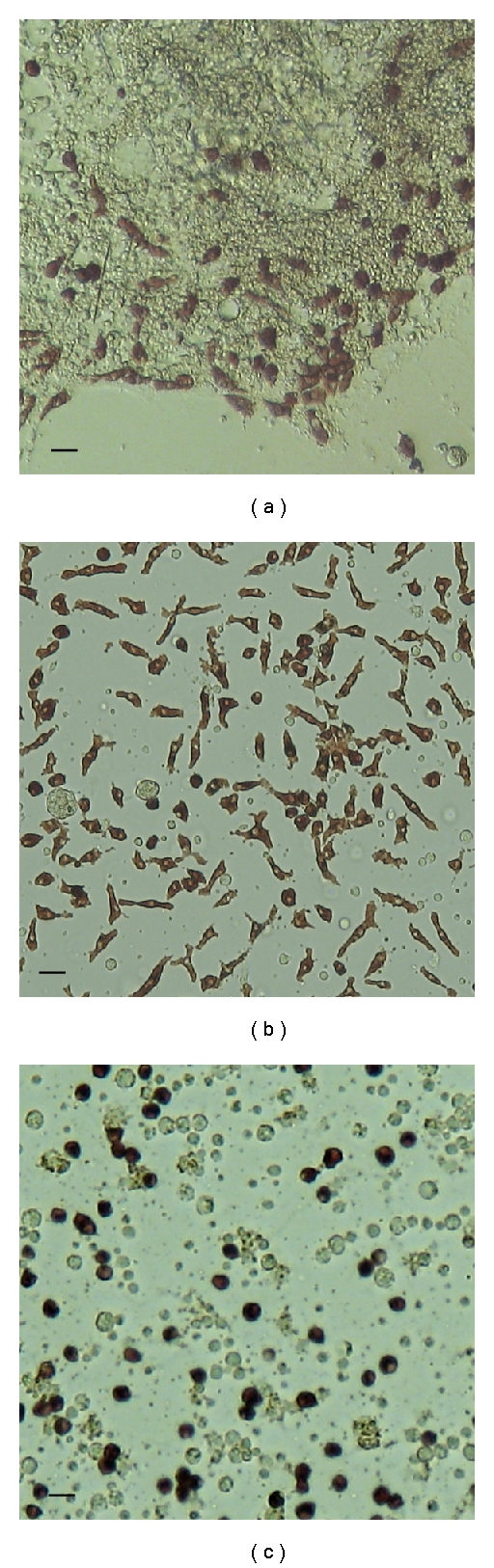
Embryonic pigment cells in a blastula-derived cell culture of the sea urchin *S. intermedius*. (a) Multilayer cell sheets (2-3 days of cultivation in seawater supplemented with 2% fetal bovine serum); (b) spread pigment cells cultivated in the coelomic fluid of control sea urchins for 3 days; (c) rounded pigment cells cultivated in the coelomic fluid of injured sea urchins for 3 days. *Bar*, 10 *μ*m.

Different conditions of cell cultivation may determine the cytodifferentiation patterns of sea urchin embryonic cells. Two days after a blastula-derived cell culture was initiated, two types of substrate-attached cells developed: epithelial and mesenchymal cells, which formed dense multilayer cell sheets (Figure 6(a)). The appearance of pigment cells among the mesenchymal elements indicated that they are derived from the secondary mesenchyme. The transfer of these cells to new dishes with fibronectin-coated coverslips resulted in intensive pigment differentiation during the following two days. It should be noted that the morphological appearance of pigment cells was dependent on the cell culture medium. If the coelomic fluid of control sea urchins was used as the medium, all the pigment cells were well attached and spread (Figure 6(b)). However, if the coelomic fluid of injured sea urchins was used as the culture medium, all the pigment cells were rounded and unspread (Figure 6(c)). Following 20 days in culture, the pigment cells maintained their morphology; however, further cell division was not detected. Cell viability was 90–95% immediately after seeding and declined to 70–75% after 20-day cultivation. 

## 4. Discussion

Marine organisms passed through the long path of evolution and adaptation, and this is reflected in the peculiarities of their biosynthesis and metabolism. It is known that the transcription factor glial cells missing (SpGCM) is required for the activation of transcription for pigment cell-specific differentiation genes; the onset of transcription of these genes occurs a few hours after the activation of *Spgcm *(12 hours) [20]. Phylogenomic studies have suggested that some animal genomes (sea urchins, birds, and fish) possess a previously unidentified group of *pks* genes in addition to *fas *genes used in fatty acid metabolism. These *pks* genes in the chicken, fish, and sea urchin genomes do not appear to be closely related to any other animal or fungal genes and instead are closely related to *pks* genes from the slime mold *Dictyostelium *and eubacteria [24].

Our results agree with the data of Calestani with colleagues that showed that the *pks *genes are expressed in sea urchin pigment cells beginning from the blastula stage and that this expression is maintained throughout the pluteus stage [21]. The level of *pks* transcripts has been found to be highest at the gastrula stage and then gradually decreases. The addition of shikimic acid (0.1 mM and 0.5 mM), a precursor of naphthoquinone pigments, to zygotes and embryos was shown to increase the expression of the *pks* and *sult* genes. The addition of lower concentrations of shikimic acid to sea urchin zygotes did not influence the larval developmental stages. However, the addition of 0.5 mM and 2.0 mM shikimic acid to the blastula and gastrula embryos resulted in a marked slowdown of normal development or in larval growth inhibition, respectively.

As shown by Kominami [25], pigment cells differentiate in embryos treated with aphidicolin, a specific inhibitor of DNA polymerase alpha although gastrulation and successive morphogenesis are blocked due to the absence of cell divisions and DNA synthesis. The number of pigment cells observed in aphidicolin-treated embryos increased as the treatment was initiated at later time points (from 9, 10, 12, 16, and 24 h of development) [25]. Pigment cells can be induced even from animal blastomeres at the 8-cell stage or mesomeres at the 16-cell stage, if the blastomeres are treated with LiCl [26, 27]. These data indicate the possible existence of an inductive signal for the specification of the pigment cell lineage.

Using dissociated sea urchin embryos transfected with the yeast gene *gal4, *we have previously shown that the absorption spectrum of red-brown pigments extracted from the cultured cells coincides with that of echinochrome [28]. The number of cells containing the red-brown pigments in two-month-old cell culture reached 50–60%, while the number of naphthoquinone pigments in these cells, as calculated per one cell [29], increased 9-10-fold [28] compared to the cells of normal plutei *in vivo* [5]. Here, we continued the studies of the differentiation process of sea urchin pigment cells in culture and developed conditions for the promotion of pigment cell differentiation without transfection of sea urchin embryos with foreign genes. Many pigmented cells formed and showed spread morphology similar to pigment cells embedded in the embryonic or larval ectoderm [16, 29]. However, there is no cell division in these cultures. Today, only cells of developmental anomalies in sea urchin embryos transformed by the yeast *gal4* gene [30], and malignant mussel hemocytes [31] have been reported to be involved in active proliferation. 

 We have found the specific effect of the coelomic fluid of control and injured sea urchins on the morphology of cultivated pigment cells. The origin of this phenomenon is unclear. We failed to develop a potential permanent cell line; however, the results obtained allow us to assert that the culture conditions used promote pigment cell differentiation and can be useful for studying sea urchin pigment cells. The technology of directed differentiation of marine invertebrate embryonic cells *in vitro* opens the pathway for solution-applied tasks, including the generation of cell cultures that produce complex bioactive compounds with therapeutic potential.
